# Critical outcomes to be included in the Core Outcome Set for nutritional intervention studies in older adults with malnutrition or at risk of malnutrition: a modified Delphi Study

**DOI:** 10.1038/s41430-024-01444-6

**Published:** 2024-05-23

**Authors:** Nuno Mendonça, Christina Avgerinou, Sibel Çavdar, Tommy Cederholm, Alfonso J. Cruz-Jentoft, Gabriel Torbahn, Cornel Sieber, Hanna M. Siebentritt, Eva Kiesswetter, Dorothee Volkert, Marjolein Visser

**Affiliations:** 1 EpiDoC Unit, Comprehensive Health Research Centre, NOVA Medical School, Universidade Nova de Lisboa, Lisbon, Portugal; 2 Centre for Ageing Population Studies, Research Department of Primary Care and Population Health, University College London, London, UK; 3 Division of Geriatrics, Department of Internal Medicine, Izmir City Hospital, Izmir, Türkiye; 4 Department of Public Health and Caring Sciences, Clinical Nutrition and Metabolism, Uppsala University, Uppsala, Sweden; 5 Theme Inflammation and Aging, Karolinska University Hospital, Stockholm, Sweden; 6 Servicio de Geriatría, Hospital Universitario Ramón y Cajal (IRYCIS), Madrid, Spain; 7 Department of Pediatrics, Paracelsus Medical University, Nürnberg, Germany; 8 Institute for Biomedicine of Aging, Friedrich-Alexander-Universität Erlangen-Nürnberg, Nürnberg, Germany; 9 Institute for Evidence in Medicine, Medical Center-University of Freiburg, Faculty of Medicine, University of Freiburg, Freiburg, Germany; 10 Department of Health Sciences, Faculty of Science, Vrije Universiteit Amsterdam, Amsterdam Public Health Research Institute, Amsterdam, The Netherlands

**Keywords:** Malnutrition, Geriatrics, Nutrition

## Abstract

**Introduction:**

As part of the development of an agreed minimum set of outcomes or Core Outcome Set (COS) for future nutritional intervention trials in older adults with malnutrition or at risk of malnutrition, this work reports on the Delphi surveys and final consensus.

**Methods:**

Outcomes from a scoping review were incorporated into a two-round Delphi survey. Researchers and healthcare professionals experienced in malnutrition in older adults were invited to take part in an online survey to rate 38 selected outcomes on a nine-point Likert scale ranging from ‘not important’ to ‘critical’ for their setting (community, hospital, or long-term care). Consensus for inclusion was reached when ≥75% (or ≥60% if a patient-reported outcome) of the participants scored the outcome as ‘critical’ and <15% as ‘not important’. Resulting outcomes were voted for inclusion or exclusion in the COS in a final online consensus meeting.

**Results:**

Ninety-three and 72 participants from diverse professional backgrounds and countries participated in the 1st and 2nd Delphi round, respectively. After both rounds eleven outcomes met the inclusion criteria, largely irrespective of setting. Fifteen participants, representing academia, health care, health policy, industry, and PPI, voted in a final online consensus meeting resulting in ten outcomes: malnutrition status, dietary intake, appetite, body weight or BMI, muscle strength, muscle mass, functional performance, functional limitations, quality of life, and acceptability of the intervention.

**Conclusions:**

Ten outcomes will form the COS which is intended to be used by the scientific community in all future nutritional intervention studies for older adults with malnutrition or at risk of malnutrition. The subsequent phase will establish the appropriate methods to measure these outcomes.

## Background

Malnutrition is widespread, its prevalence ranging from 3–15% amongst older adults living in the community, 18–29% amongst older long-term care residents, and 22% amongst hospitalized older adults, while malnutrition risk ranges from 27–48% in the community, 48–52% in long-term care and 46–53% in the hospital, depending on the geographical location and tool used [1–3]. Malnutrition is associated with severe consequences such as diminished quality of life [4], functional decline [5], hospital re-admission and early death [6, 7], and higher social and health care costs [8]. Malnutrition is of great concern to healthcare professionals and governments alike, but several knowledge gaps in its prevention and treatment still exist [9–12]. Unfortunately, the diversity of outcomes and methods in trials assessing the effectiveness of nutritional interventions complicates comparisons and combining data, which leads to considerable resource wastage.

A core outcome set (COS) is an agreed minimum set of outcomes that should be measured and reported in all trials of a specific disease or trial population [13]. Incorporating COS in all nutritional intervention trials on malnutrition has three main benefits: (a) to enable easier result comparison across trials, including aggregating summary results or individual participant data, (b) to optimise use of resources, and c) to reduce information bias and selective reporting of outcomes. Ultimately, the use of a COS supports clinical decision making by identifying the most effective approach for treating and preventing malnutrition in older adults [13]. There is currently no COS for any nutritional intervention studies in older adults mainly due to the lack of awareness of the existence and need of a COS, and the methodological rigour behind the development process [14].

The overarching aim of this project is to develop a COS for future nutritional intervention trials in older adults with malnutrition or at risk of malnutrition. Following from the scoping review for commonly used outcomes in previous trials [15], this study reports on the results of the Delphi study and final consensus meeting.

## Methods

### General description of the project

This project was initiated within the special interest group Nutrition of the European Geriatric Medicine Society (EuGMS) and consists of five phases. *Phase 1—*a scoping review [15], *Phase 2—*online Delphi surveys and validation by patient and public involvement (PPI) representatives, *Phase 3—*final consensus meeting, *Phase 4—*selection of measurement properties of COS and *Phase 5—*dissemination and implementation (Fig. S1). The COMET (Core Outcome Measures in Effectiveness Trials) Initiative provides mainly two resources: a database where researchers, practitioners and patients can find COS that are both existing and in development, and methodological guidance on the development of COS [16]. The project has been registered in the COMET registry [17] and developed according to COMET guidelines [13]. More details can be found in the project protocol [18]. In *Phase 2*, all outcomes from the scoping review [15] and from additional literature search for patient reported outcomes (PROs) were reviewed by the steering group and PPI representatives, and a minimally modified list (e.g. Mitochondrial ATP production was identified from *Phase 1* but not included; program satisfaction, dietary satisfaction and acceptance of the intervention were merged into acceptability; and (re)hospitalisation was included as part of healthcare use) incorporated into an online two-round, modified Delphi survey in English. Questionnaires were developed in Google Forms. The two main differences to the original Delphi method are that there was a scoping review before hand to identify the most commonly used outcomes in nutritional intervention trials and there was a limit of Delphi rounds, as opposed to unlimited.

### Meetings with patient and public involvement (PPI) representatives

Briefly, before the survey was sent out, feedback on the list of outcomes, including PROs, was sought from three members of the public representing older adults and their caregivers. Two of the members were part of a UK-based PPI association and one was from Türkiye (Turkey) chosen by convenience from outpatient consultations in a local hospital. As a result, hydration status and eating behaviour were kept as distinct outcomes and not under “non-critical outcomes” contrary to the initial decision of the steering committee. Additionally, the list of outcomes to be rated was expanded to include self-esteem (related to confidence in one’s appearance as reflected in the descriptions on Table S1).

### Delphi survey 1st round

Researchers and healthcare professionals with ample experience in malnutrition in older adults (≥3 international, peer-reviewed scientific publications on the topic as a first or last author, or active clinical practice with experience on the topic) were invited by email between July and December 2022 to take part in an online survey to rate and motivate the rating of 38 outcomes [body weight or body mass index (BMI), body circumference(s), skinfold(s), malnutrition status, falls, frailty, mortality, healthcare use, healthcare costs, complications, health status, dysphagia severity, fatigue, weakness, self-perceived health, pain, quality of life, cognitive status, depression, anxiety, sleep disturbance, self-esteem, dietary intake, appetite, hydration status, eating behaviour, energy requirements, blood marker(s), nitrogen balance, muscle mass, muscle strength, functional performance, functional limitations, participation in social roles and activities, peak expiratory flow, bone health, acceptability or adherence of the intervention, adverse events] on a nine-point Likert scale ranging from ‘not important’ to ‘critical’. Description of each outcome is shown in Table S1. Weight loss was not included as a separate outcome because it can be calculated from body weight measured at baseline and follow-up. The steering group considered nine outcomes reported in previous trials (hunger, resting energy expenditure, eating disorder, prescription (par)enteral nutrition, chemotherapy management, grade 3–4 toxicities, self-satisfaction, physical activity, and healthy lifestyle) as ‘non-critical’. These nine outcomes were grouped together, and participants asked to agree or disagree with their exclusion and motivate their choice. Participants were also asked to indicate in which setting (community, hospital, and long-term care) and with what follow-up time [short-term (≤12 weeks) or long-term (>12 weeks)] of nutritional interventions they had most experience with or considered most relevant and rate the outcomes accordingly. Additionally, information on gender, age, and occupation was collected. Participants were randomly assigned to one of four versions of the survey with a different order of outcomes.

Consensus for inclusion in the COS was reached when ≥75% (or ≥60% if a PRO) of the participants scored the outcome as ‘critical’ and <15% as ‘not important’. Similarly, an outcome was excluded when ≥75% of the participants scored the outcome as ‘not important’ and <15% as ‘critical’.

### Delphi survey 2nd round

Those who participated in the 1st round were sent their previous ratings plus the average ratings from the 1st round via email between March and April 2023, and asked to re-rate 29 outcomes for which no consensus was reached in round 1 (body circumference(s), skinfold(s), falls, frailty, mortality, healthcare use, healthcare costs, complications, health status, dysphagia severity, fatigue, weakness, self-perceived health, pain, cognitive status, depression, anxiety, sleep disturbance, self-esteem, appetite, hydration status, eating behaviour, energy requirements, blood marker(s), nitrogen balance, muscle mass, participation in social roles and activities, peak expiratory flow, bone health). Physical activity was added as a new outcome based on feedback from round 1. Additionally, outcomes that met consensus for inclusion in the 1st round were grouped and participants were asked to agree or disagree with its inclusion and motivate their choice. Participants were randomly assigned to one of three versions of the survey with a different order of outcomes. Consensus criteria were the same as in round 1.

### Validation by PPI representatives

Older adults with malnutrition or at risk of malnutrition, older adults who experienced malnutrition in the past, and caregivers of older people with malnutrition or at risk of malnutrition were invited to list five or less preferred outcomes. Additionally, the PPI representatives were asked to agree, disagree or, neither agree nor disagree with the outcomes that had reached consensus in both Delphi rounds. The feedback was obtained in the local language.

### Final consensus meeting

A final online consensus meeting was organised with a subset of Delphi participants who participated in both rounds balanced by stakeholder group, setting, gender, country, and background or discipline, together with the steering group and PPI representatives. In addition, two representatives from the medical nutrition industry were invited. Results from the Delphi rounds and the validation by PPI representatives were presented. Final consensus was achieved when ≥70% of the 15 participants, through an anonymous vote, agreed with the inclusion of the outcome or exclusion of undecided outcomes from the 1st and 2nd round of the Delphi survey, as per the protocol [18].

### Data management, Ethical approval, and consent

Email invitations were sent to publicly available email addresses. The email addresses of participants that consented to participate in the 1st Delphi round were stored together with the survey responses to allow approaching participants for the 2nd Delphi round. After the 2nd round an anonymous id was given to each email, the key was stored in a separate encrypted file and kept until the end of the project. All data collected were stored in a password protected Universidade Nova de Lisboa’s server. Ethical approval was obtained from the Universidade Nova de Lisboa, Lisbon, Portugal (86/2022/CEFCM).

### Statistical analyses

Scores were categorised into ‘not important’ (score 1–3), ‘important but not critical’ (4–6) and ‘critical’ (7–9) as recommended [13]. Categorical data are presented as percentages (%) with the corresponding frequency (*n*). Non-difference between settings and follow-up duration was tested with chi-square using the difference between percentages, the sample size, and the *p*-value. Difference between participant ratings and the inclusion threshold of selected outcomes (60% for PROs and 75% for non-PROs) for the 1st round and the 2nd round of the Delphi survey, and final outcomes from the consensus meeting are shown as bar plots using the *ggplot2* package v3.4.2 [19]. Data management and all descriptive analyses were performed in R v4.1.2 [20].

## Results

### Socioeconomic, geographical, and professional characteristics

Of the 316 experts invited, 93 participated in the 1st Delphi round. The most common age group were the 40–49-year-olds (32%, *n* = 30) while the distribution of other age groups was similar, except for an underrepresentation of 20–29-year-olds (3%, *n* = 3) (Table 1). Most participants were women (77%, *n* = 72), from Europe (72%, *n* = 67) followed by Asia (14%, *n* = 13), and although 27 countries were represented, most hailed from the Netherlands, Türkiye, UK, and Portugal. Two-thirds (66%, *n* = 62) of the participants were either geriatricians or researchers while 16% (*n* = 15) were dietitians or nutritionists. A little more than half of the participants worked mainly in the hospital (55%, *n* = 51), 36% (*n* = 33) in the community and 10% (*n* = 9) in long-term care, while approximately half (48%, *n* = 45) responded that the main duration of follow-up of nutritional interventions in their setting was <12 weeks. The response rate for the 2nd Delphi round was 77.4% (*n* = 72). The distribution of the characteristics was similar between the 1st and 2nd rounds, and importantly, Europe was still overrepresented in the latter (75%, *n* = 54) (Table 1).

**Table 1:** Sociodemographic characteristics of participants of the 1st and 2nd Delphi Survey round.

	Delphi 1st round (*n* = 93)	Delphi 2nd round (*n* = 72)
*Age, % (n)*		
20–29 y	3 (3)	3 (2)
30–39 y	23 (21)	22 (16)
40–49 y	32 (30)	29 (21)
50–59 y	19 (18)	18 (13)
60+ y	23 (21)	28 (20)
Men, % (*n*)	23 (21)	25 (18)
*Continent, % (n)*		
America	8 (7)	7 (5)
Asia	14 (13)	12 (9)
Europe	72 (67)	75 (54)
Oceania	7 (6)	6 (4)
*Occupation, % (n)*		
Dietitian/ Nutritionist	16 (15)	17 (12)
Geriatrician	33 (31)	32 (23)
Internist	9 (8)	7 (5)
Researcher	33 (31)	36 (26)
Other	9 (8)	8 (6)
*Workplace, % (n)*		
Hospital	42 (39)	39 (28)
University	47 (44)	51 (37)
Other	11 (10)	10 (7)
*Setting, main, % (n)*		
Community	36 (33)	39 (28)
Hospital	55 (51)	53 (38)
Long-term care	10 (9)	8 (6)
*Follow-up duration, main, % (n)*		
<12 weeks	48 (45)	47 (34)
≥12 weeks	52 (48)	53 (38)
*Survey version, % (n)*		
One	24 (22)	32 (23)
Two	24 (22)	36 (26)
Three	26 (24)	32 (23)
Four	27 (25)	n/a

Note: Main setting refers to the setting where most of the work of the participants concerning older adults with malnutrition and at risk took place. Equally, main follow-up duration refers to the time that was most common/preferred for nutritional interventions in the participant’s setting. *n/a* not applicable, *y* years.

### Delphi survey 1st round

The exclusion of nine non-critical outcomes was agreed by 71% (*n* = 66) of the participants but 20% (*n* = 19) considered that physical activity should be rated in the 2nd round, which was done. For all other outcomes, only ≤4 participants did not agree with their exclusion.

**Fig. 1:**
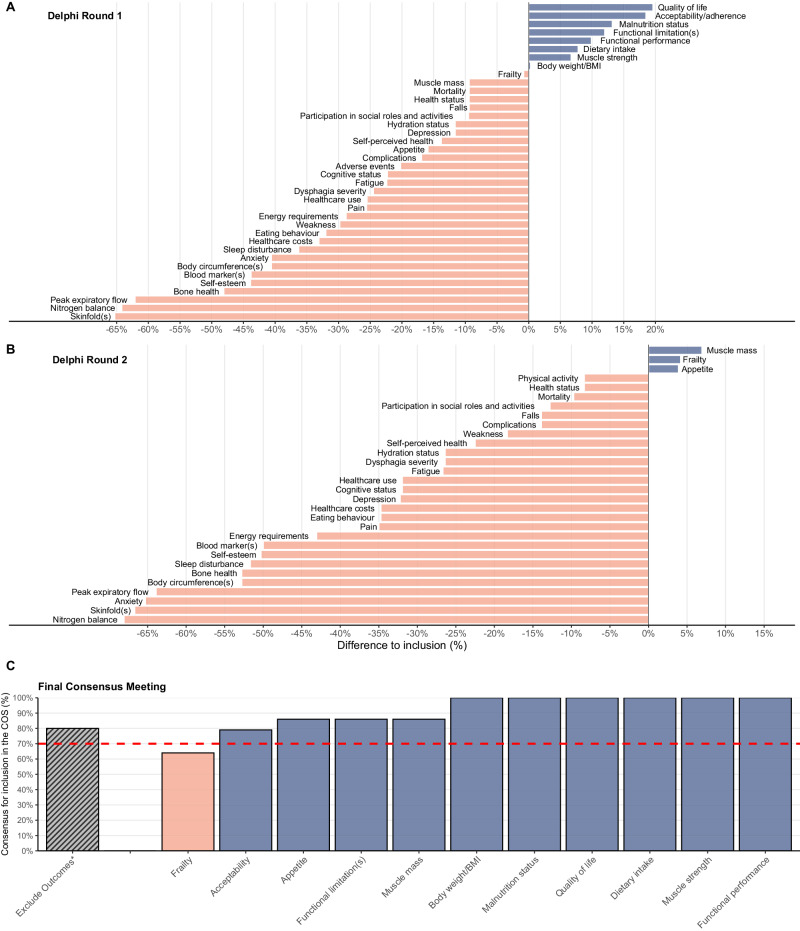
Difference between participants’ rating and the inclusion threshold of selected outcomes of the Delphi survey and final consensus. **A** Difference of the participants’ rating to the threshold of inclusion of selected outcomes (60% for PROs and 75% for non-PROs) for the 1st round (*n* = 93) and **B** the 2nd round of the Delphi survey (*n* = 72). Adverse events were excluded, and physical activity included in the 2nd round of the survey. **C** Final consensus meeting: percentage of participants that agreed to the inclusion of these outcomes in the Malnutrition COS (*n* = 15). Outcomes with ≥70% agreement were included in the COS.  and  bars mean outcomes that were above and below consensus threshold according to protocol, respectively. *Outcomes to be voted together for exclusion included Body circumference(s), Skinfold(s), Mortality, Healthcare use, Healthcare costs, Complications, Health status, Dysphagia severity, Fatigue, Weakness, Self-perceived health, Pain, Cognitive status, Depression, Anxiety, Sleep disturbance, Self-esteem, Hydration status, Eating behaviour, Energy requirements, Blood marker(s), Nitrogen balance, Participation in social roles and activities, Peak expiratory flow, Bone health, Falls, Physical activity. It is worth noting that weight loss can be calculated from assessing body weight at baseline and follow-up. BMI body mass index, COS core outcome set.

In this round, ≥75% of participants rated malnutrition status (88%), dietary intake (83%), body weight or BMI (75%), muscle strength (82%) and functional performance (85%) as critical, while ≥60% rated the PROs functional limitations (72%), quality of life (80%), and acceptability or adherence of the intervention (79%) as critical to be included in the COS (Fig. 1A, Table S2).

No single outcome was considered sufficiently unimportant, warranting its re-rating in the subsequent Delphi round (≥75% not important and <15% critical for non-PROs and ≥60% not important and <15% critical for PROs). Therefore, because it was unclear which outcomes to exclude, participants were asked to re-rate these in a 2nd Delphi survey round.

Rating of outcomes from the 1st round of the Delphi survey were largely similar by setting (community *n* = 33, hospital *n* = 51 and long-term care *n* = 9) (Table S3) and duration of follow-up (<12 weeks *n* = 45, ≥12 weeks *n* = 48) (Table S3). However, there were some notable exceptions, that although not warranting a different COS by setting are important to mention. For example, in the hospital setting ≥75% of the participants (77%) rated mortality as a critical outcome to be included in the COS but only less than half (49%) did so in the community setting. Similarly, 71% of the participants from the hospital setting rated complications as critical while only 39% did so in the community. There were few participants who indicated long-term care as their main setting (*n* = 9), but it seemed that dysphagia severity (*n* = 8) and hydration status (*n* = 7) were rated as more important than in other settings (Table S3).

### Delphi survey 2nd round

Adverse events were excluded for re-rating in the 2nd round since the steering group considered that reporting adverse events is already mandatory for any trial, and therefore, did not need to be included in a specific COS. Furthermore, for the same reasons and because the concepts were too different, adherence to the intervention was removed from the outcome acceptability.

Almost all participants (99%) in the 2nd round agreed that malnutrition status, body weight or BMI, functional performance, dietary intake, muscle strength, functional limitations, quality of life and acceptability of intervention should be included in the COS. One participant disagreed with the inclusion of acceptability of intervention and quality of life (Fig. 1B, Table S3).

On top of these, ≥75% of participants re-rated muscle mass (82%) and frailty (79%) as being critical for inclusion in the COS, and ≥60% re-rated appetite (64%) as a PRO that was critical to be included in the COS.

### Validation by PPI representatives

Five PPI representatives (three adults aged 80+ years: 1 malnourished and 2 at high risk, and two informal care givers) from the Netherlands, Türkiye and Portugal considered well-being, looking good, walking without help and fatigue, weight regain, strength, physical capacity, being able to do sports and memory as an important outcome for them. The steering group concluded that these outcomes largely reflected the results of the two Delphi rounds except for memory and looking better. However, cognitive status was rated low in both Delphi rounds. It was discussed that looking good might have some overlap with self-perceived health (rated low), self-esteem (rated low), and quality of life (included), and that this type of outcome would likely become more important in the future and would require a specifically designed study.

The PPI representatives also broadly agreed with the outcomes derived from the 2 Delphi rounds. However, there were five neutral votes (1xmalnutrition status, 1× muscle mass, 2× frailty and 1× acceptability of intervention) and one negative vote for functional performance.

### Final consensus meeting

The final consensus meeting took place online on the 27th of July 2023 with 15 participants present plus the chair who abstained: 2 PPI representatives (where one joined halfway through the meeting), 6 steering group members, 5 participants from both Delphi rounds (selected for their involvement in health policy or being a dietitian) and 2 external guests from the medical nutrition industry.

Participants were asked if they agreed with the exclusion of the undecided outcomes from both Delphi rounds (i.e., body circumference(s), skinfold(s), mortality, healthcare use, healthcare costs, complications, health status, dysphagia severity, fatigue, weakness, self-perceived health, pain, cognitive status, depression, anxiety, sleep disturbance, self-esteem, hydration status, eating behaviour, energy requirements, blood marker(s), nitrogen balance, participation in social roles and activities, peak expiratory flow, bone health, falls and physical activity). Less than 70% of the participants (64%) agreed with the exclusion of these outcomes so no consensus was reached. Those that did not agree with the exclusion, did so for health care costs, or self-rated health, or hydration status, or physical activity, or body circumference(s), or adverse events, or complications, or mortality. This was followed by a group discussion. Healthcare costs were considered particularly important, but participants decided these should not be mandatory for smaller trials but advisable for larger trials where a cost-effectiveness analysis may be done. Self-rated health was considered to be at least partly reflected in quality of life and therefore unnecessary to be further included in the COS. The participants considered hydration status of lower importance considering the effects of malnutrition treatment but acknowledged that nutritional interventions are not only about foods and nutrients. Inclusion of physical activity in the COS was not supported since physical activity was rated low in the 2nd Delphi round, and consensus meeting participants considered that individuals could be physically active or inactive regardless of nutritional status. Body circumference was mentioned as being a simple and feasible measure of muscle mass. However, this comment referred to *how* an outcome should be measured, which is not relevant for this phase of the COS development. After the group discussion, 80% of the participants (out of 15 since both PPI representatives were now present) re-voted to exclude all undecided outcomes from the COS (Fig. 1C).

Outcomes that had reached consensus in at least one of the Delphi rounds were all voted in for inclusion in the final COS, with the exception of frailty as it did not reach ≥70% consensus in the final voting since the group considered that components of frailty largely overlapped with other COS outcomes (Fig. 1C).

## Discussion

### Main findings

Ten outcomes met the criteria for inclusion in the COS after both Delphi rounds and the final consensus meeting: malnutrition status, dietary intake, appetite, body weight or BMI, muscle strength, muscle mass, functional performance, functional limitations, quality of life, and acceptability of the intervention. There were not enough significant differences between settings that warranted the creation of a setting-specific COS. These ten selected outcomes are intended to be used by the scientific community in any future trials assessing the effectiveness of nutritional interventions in older adults with malnutrition and those at risk.

A COS is an agreed minimum set of outcomes that should be measured and reported in all trials of a specific disease or trial population. However, it does not limit the number of outcomes included, provided the study follows the CONSORT-Outcomes 2022 extension, namely defining primary and secondary outcomes, reporting results for all outcomes and identifying any outcome that was not prespecified in the protocol or registry if appropriate [21]. Therefore, nutritional intervention trials on older adults with malnutrition or at risk of malnutrition which have specific research questions that can only be answered by additional outcomes can and should do so. For example, studies in the hospital setting can include mortality as an outcome on top of the existing COS.

Furthermore, this COS was developed for trials on older adults with malnutrition and at risk of malnutrition, but the steering group considers that it can also be useful when the aim of the intervention is prevention of malnutrition in participants that are neither at risk nor malnourished. The steering group also considers that, although the scoping review was done for trials only (*Phase 1* of this project—Fig. S1) [15], this COS is also applicable in routine care, clinical audits, and study types other than clinical trials, such as longitudinal cohorts (except for acceptability of the intervention in an observational study or in routine care when there is no intervention).

The discussions of the steering group and consensus meeting group also highlighted the importance of all nutritional intervention trials reporting any adverse events and adherence of the intervention. Although adverse events and adherence are not part of the COS since they are already mandatory for any trial, the steering group considered that these are not always reported, and their importance should be noted. Furthermore, while a major focus of nutritional interventions in older adults with malnutrition or at risk of malnutrition is on increasing energy and nutrient intake, attention should also be paid to fluid intake to optimize hydration status. Finally, for larger trials testing novel interventions, participants of the consensus meeting advised on the inclusion of health care costs as an outcome to enable cost-effectiveness analyses.

While up until now the focus was on establishing *which* outcome should be included in the COS, culminating in the selection of ten outcomes, the next phase will establish *how* these should be measured. The possible assessment methods for these ten outcomes have been identified in the scoping review [15] of *Phase 1* and will be evaluated by the core or steering group according to their measurement properties [22]. The core or steering group will be allowed to add relevant assessment methods not listed in the scoping review and any newly developed assessment methods not yet cited in publications.

### Strengths and weaknesses

Despite an increased effort during recruitment, only few experts representing the long-term care setting were included. As a result, differences between long-term care and other settings should be taken with caution. This means that this COS may not be applicable to this setting. However, on top of extra outcomes (e.g., dysphagia severity and hydration status), the few participants from long-term care identified the same outcomes as being critical to be included in the COS as those from other settings. Therefore, this COS may be a good starting point for the development of a future long-term care-specific COS.

Furthermore, more than 2/3 of the participants in the 1st and 2nd Delphi round were from Europe which may limit some generalizability of the COS outside of Europe. However, there were very little differences when stratifying results from both Delphi rounds by participants from Europe and elsewhere (Table S4). An important limitation was that no older adults with malnutrition and at risk took part in the two online Delphi rounds. The steering group was concerned that older adults with malnutrition in the hospital or long-term care would often suffer from functional and cognitive limitations, acute disease(s), insufficient digital literacy, and/or language barriers to complete an online survey in English language. As an alternative, older adults and caregivers were present at several stages as PPI representatives, including the final consensus meeting, more PROs were added to the list of outcomes to be rated after the scoping review, and PROs had a laxer COS inclusion criterion. Notwithstanding, future COS efforts should consider alternative methods to circumvent these limitations, such as the option of a hybrid format and multiple translations of the Delphi survey.

The aim, as per protocol, was to recruit 200 participants for the Delphi survey to account for attrition between the 1st and 2nd round and end up with 50 participants per setting (community, hospital and long-term-care), balanced between clinical practice and academia, gender and geographical location [18]. However, despite extending the recruitment period, using other sources to find experts (e.g., checking emailing lists from other special interest groups in other societies), asking for referrals of healthcare professionals, and sending reminders, we did not reach the intended total number of participants. This may have made the study results less robust. However, the rigorous methodology, the possibility of participants to propose new outcomes beyond the original 38 and the good distribution of characteristics between participants make this less likely. Other COS have used similar sample sizes [23, 24]. There was an attrition of 23% from the 1st (*n* = 93) to the 2nd round (*n* = 72) of the Delphi survey. However, sociodemographic characteristics between Delphi rounds were virtually similar which makes selection bias between rounds less likely to have happened (Table 1). As important strengths the project was registered in the COMET Initiative registry of COS [17], developed according to the COMET guidelines [13] and the protocol was published beforehand [18]. Furthermore, relevant stakeholder groups were represented in the final consensus meeting.

## Conclusions

The COS consists of ten outcomes: malnutrition status, dietary intake, appetite, body weight or BMI, muscle strength, muscle mass, functional performance, functional limitations, quality of life, and acceptability of the intervention. The COS will guide the selection of outcomes in future nutritional intervention studies for older adults with malnutrition or at risk of malnutrition. The subsequent phase will establish the appropriate methods for measuring these outcomes.

## Availability of data and materials

An anonymized dataset of both Delphi rounds and accompanying code for data management and analysis can be shared in case of justified interest upon request to nuno.mendonca@nms.unl.pt.

### Supplementary information

Critical outcomes to be included in the Core Outcome Set for nutritional intervention studies in older adults with malnutrition or at risk of malnutrition: a modified Delphi Study
