# Supplementary material for: Critical outcomes to be included in the Core Outcome Set for nutritional intervention studies in older adults with malnutrition or at risk of malnutrition: a modified Delphi Study
Source: Eur J Clin Nutr. 2024 May 23;78(8):663–9. doi: 10.1038/s41430-024-01444-6 (PMC11300301; doi:10.1038/s41430-024-01444-6)
Supplement: Supplementary file 1 — Critical outcomes to be included in the Core Outcome Set for nutritional intervention studies in older adults with malnutrition or at risk of malnutrition: a modified Delphi Study [file 41430_2024_1444_MOESM1_ESM.docx]

**Supplementary Material**

**Critical outcomes to be included in the Core Outcome Set for nutritional intervention studies in older adults with malnutrition or at risk of malnutrition: a modified Delphi Study**

Nuno Mendonça^1^, Christina Avgerinou^2^, Sibel Çavdar^3^, Tommy Cederholm^4,5^, Alfonso J. Cruz‑Jentoft^6^, Gabriel Torbahn^7^, Cornel Sieber^8^, Hanna M. Siebentritt^8^, Eva Kiesswetter^9^, Dorothee Volkert^8^, Marjolein Visser^10^

^1^ EpiDoC Unit, Comprehensive Health Research Center, NOVA Medical School, Universidade Nova de Lisboa, Portugal

^2^ Centre for Ageing Population Studies, Research Department of Primary Care and Population Health, University College London, London, UK

^3^ Division of Geriatrics, Department of Internal Medicine, Ege University Hospital, Izmir, Turkey

^4^ Department of Public Health and Caring Sciences, Clinical Nutrition and Metabolism, Uppsala University, Uppsala, Sweden

^5^ Theme Inflammation and Aging, Karolinska University Hospital, Stockholm, Sweden

^6^ Servicio de Geriatría, Hospital Universitario Ramón y Cajal (IRYCIS), Madrid, Spain

^7^ Department of Pediatrics, Paracelsus Medical University, Nürnberg, Germany

^8^ Institute for Biomedicine of Aging, Friedrich-Alexander-Universität Erlangen-Nürnberg, Nürnberg, Germany

^9^ Institute for Evidence in Medicine, Medical Centre-University of Freiburg, Faculty of Medicine, University of Freiburg, Freiburg, Germany

^10^ Department of Health Sciences, Faculty of Science, Vrije Universiteit Amsterdam, Amsterdam Public Health Research Institute, Amsterdam, The Netherlands

Corresponding author: Nuno Mendonça, email: [nuno.mendonca@nms.unl.pt](mailto:nuno.mendonca@nms.unl.pt)

**Figure S1.** Overview of the project phases (phase 1-5) highlighting phase 2 and 3 of the current study.


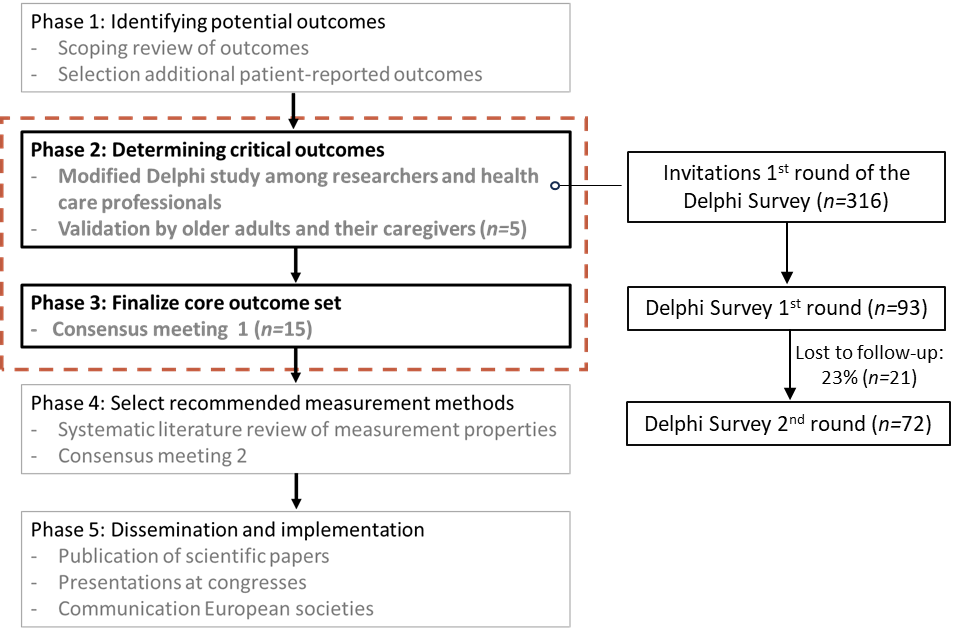


Note: Fourteen participants rated the outcomes during half the consensus meeting but one more PPI representative (*n=*15) joined for the re-voting of the excluded outcomes.

**Table S1.** Outcomes and definitions used in the Delphi survey and consensus meeting.

| **Outcome** | **Definitions** | **Delphi round** |
| --- | --- | --- |
| **Dietary Intake** | assessment of the energy and nutrient intake | 1^st^ |
| **Appetite** | feeling that you want to eat food | 1^st^, 2^nd^ |
| **Hydration status** | the balance between water input and output measured by any method | 1^st^, 2^nd^ |
| **Eating behaviour** | broad term that encompasses food choice and motives, feeding practices and dieting (not disorders) | 1^st^, 2^nd^ |
| **Energy requirements** | energy needed to balance energy expenditure in order to maintain body size, body composition and a level of necessary and desirable physical activity consistent with long-term good health measured by any method | 1^st^, 2^nd^ |
| **Body weight or Body mass index (BMI)** | measurement of body weight or BMI | 1^st^ |
| **Body circumference(s)** | measurement of body circumference(s), such as calf, mid-upper arm, or thigh circumference | 1^st^, 2^nd^ |
| **Skinfold(s)** | measurement of thickness of (a) skinfold(s), such as the triceps, sub-scapula, supra-iliac or abdominal skinfold | 1^st^, 2^nd^ |
| **Malnutrition status** | assessment of nutritional status using any validated assessment or screening tool | 1^st^ |
| **Blood marker(s)** | such as albumin, pre-albumin, transferrin, C-reactive protein, total cholesterol, haemoglobin, and creatinine | 1^st^, 2^nd^ |
| **Nitrogen balance** | measurement of nitrogen input minus nitrogen output | 1^st^, 2^nd^ |
| **Muscle mass** | measurement of muscle mass (or related measures such a fat-free mass or lean body mass) by any methodology | 1^st^, 2^nd^ |
| **Muscle strength** | measurement of muscle strength by any instrument and with any protocol | 1^st^ |
| **Functional performance** | objective measurement of functional performance using one or more timed performance tests | 1^st^ |
| **Functional limitation(s)** | assessment of self-reported functional limitations by activities of daily living, and/or instrumental activities of daily living, and/or mobility limitations | 1^st^ |
| **Participation in social roles and activities** | doing regular leisure activities with others, usual work, family activities and activities with friends that someone wants to do | 1^st^, 2^nd^ |
| **Peak expiratory flow** | maximum flow rate generated during a forceful exhalation, starting from full inspiration | 1^st^, 2^nd^ |
| **Bone health** | Any measure of bone health (not falls) such as bone mineral density | 1^st^, 2^nd^ |
| **Falls** | an event which results in a person coming to rest inadvertently on the ground or floor or other lower level | 1^st^, 2^nd^ |
| **Frailty** | geriatric syndrome characterised by increased vulnerability to stressors as measured by the frailty phenotype, the accumulated deficits, or any other indexes. This outcome does not include having functional limitations or disability as these are considered separate outcomes. | 1^st^, 2^nd^ |
| **Mortality** | measurement of rate of death | 1^st^, 2^nd^ |
| **Health care use** | quantification of the use of services by people for the purpose of preventing and curing health problems, promoting maintenance of health and well-being, or obtaining information about one's health status and prognosis | 1^st^, 2^nd^ |
| **Health care costs** | actual cost of services used by people for the purpose of preventing and curing health problems, promoting maintenance of health and well-being, or obtaining information about one's health status and prognosis | 1^st^, 2^nd^ |
| **Complications** | secondary diseases or conditions aggravating an already existing one | 1^st^, 2^nd^ |
| **Health status** | the relative level of wellness and illness of a person, taking into account the presence of biological or physiological dysfunction, morbidity, symptoms, and functional impairment | 1^st^, 2^nd^ |
| **Dysphagia severity** | Severity/intensity of swallowing problems | 1^st^, 2^nd^ |
| **Fatigue** | an overall feeling of tiredness or lack of energy | 1^st^, 2^nd^ |
| **Weakness** | self-reported state of feeling/being weak | 1^st^, 2^nd^ |
| **Pain** | self-reported pain interference with day-to-day activities | 1^st^, 2^nd^ |
| **Self-perceived health** | self-reported rating of general health status | 1^st^, 2^nd^ |
| **Quality of life** | degree to which a person is healthy, comfortable, and able to participate in or enjoy life events, as assessed by any questionnaire or information chart | 1^st^ |
| **Cognitive status** | assessment of cognitive status, including objective and subjective methods using a questionnaire or a test or any other method | 1^st^, 2^nd^ |
| **Depression** | assessment of depressive symptoms using a questionnaire, a clinical evaluation, or any other method | 1^st^, 2^nd^ |
| **Anxiety** | assessment of level of anxiety using a questionnaire, a clinical evaluation, or any other method | 1^st^, 2^nd^ |
| **Sleep disturbance** | self-reported problems sleeping | 1^st^, 2^nd^ |
| **Self-esteem** | Confidence in one's own appearance, worth and abilities | 1^st^, 2^nd^ |
| **Acceptability of the nutritional intervention** | any measure providing information on how acceptable the intervention is for the older person | 1^st^ |
| **Adverse events** | any untoward medical problem in a patient receiving the intervention and which does not necessarily have a causal relationship with the intervention | 1^st^ |
| **Physical activity** | any bodily movement produced by skeletal muscle that requires energy expenditure including during leisure time, for transport to get to and from places, or as part of a person’s work, such as walking, recreational activities, exercise, housework, gardening, etc | 2^nd^ |

Note: weight loss can be calculated from body weight measured at baseline and follow-up.

**Table S2.** Participants’ rating of the outcomes (not important, important but not critical and critical) for the 1^st^ and 2^nd^ Delphi Survey round

|  | **Delphi 1^st^ round** % (n) | **Delphi 2^nd^ round** % (n) |
| --- | --- | --- |
| **Number of participants** | 93 | 72 |
| **Body weight/ BMI** |  |  |
| Not important | 7.5 (7) | n/a |
| Important but not critical | 17.2 (16) | n/a |
| Critical | 75.3 (70) | n/a |
| **Body circumference(s)** |  |  |
| Not important | 22.6 (21) | 18 (13) |
| Important but not critical | 43.0 (40) | 60 (43) |
| Critical | 34.4 (32) | 22 (16) |
| **Skinfold(s)** |  |  |
| Not important | 43.0 (40) | 36 (26) |
| Important but not critical | 47.3 (44) | 56 (40) |
| Critical | 9.7 (9) | 8 (6) |
| **Malnutrition status** |  |  |
| Not important | 2.2 (2) | n/a |
| Important but not critical | 9.7 (9) | n/a |
| Critical | 88.2 (82) | n/a |
| **Falls** |  |  |
| Not important | 4.3 (4) | 6 (4) |
| Important but not critical | 30.1 (28) | 33 (24) |
| Critical | 65.6 (61) | 61 (44) |
| **Frailty** |  |  |
| Not important | 4.3 (4) | 1 (1) |
| Important but not critical | 21.5 (20) | 19 (14) |
| Critical | 74.2 (69) | 79 (57) |
| **Mortality** |  |  |
| Not important | 4.3 (4) | 7 (5) |
| Important but not critical | 30.1 (28) | 28 (20) |
| Critical | 65.6 (61) | 65 (47) |
| **Healthcare use** |  |  |
| Not important | 6.5 (6) | 6 (4) |
| Important but not critical | 44.1 (41) | 51 (37) |
| Critical | 49.5 (46) | 43 (31) |
| **Healthcare costs** |  |  |
| Not important | 10.8 (10) | 7 (5) |
| Important but not critical | 47.3 (44) | 53 (38) |
| Critical | 41.9 (39) | 40 (29) |
| **Complications** |  |  |
| Not important | 6.5 (6) | 4 (3) |
| Important but not critical | 35.5 (33) | 35 (25) |
| Critical | 58.1 (54) | 61 (44) |
| **Health status** |  |  |
| Not important | 1.1 (1) | 4 (3) |
| Important but not critical | 33.3 (31) | 29 (21) |
| Critical | 65.6 (61) | 67 (48) |
| **Dysphagia severity** |  |  |
| Not important | 17.2 (16) | 14 (10) |
| Important but not critical | 32.3 (30) | 38 (27) |
| Critical | 50.5 (47) | 49 (35) |
| **Fatigue** |  |  |
| Not important | 16.1 (15) | 10 (7) |
| Important but not critical | 46.2 (43) | 57 (41) |
| Critical | 37.6 (35) | 33 (24) |
| **Weakness** |  |  |
| Not important | 8.6 (8) | 11 (8) |
| Important but not critical | 46.2 (43) | 47 (34) |
| Critical | 45.2 (42) | 42 (30) |
| **Self perceived health** |  |  |
| Not important | 6.5 (6) | 8 (6) |
| Important but not critical | 47.3 (44) | 54 (39) |
| Critical | 46.2 (43) | 38 (27) |
| **Pain** |  |  |
| Not important | 22.6 (21) | 24 (17) |
| Important but not critical | 43.0 (40) | 51 (37) |
| Critical | 34.4 (32) | 25 (18) |
| **Quality of life** |  |  |
| Not important | 1.1 (1) | n/a |
| Important but not critical | 19.4 (18) | n/a |
| Critical | 79.6 (74) | n/a |
| **Cognitive status** |  |  |
| Not important | 9.7 (9) | 15 (11) |
| Important but not critical | 37.6 (35) | 42 (30) |
| Critical | 52.7 (49) | 43 (31) |
| **Depression** |  |  |
| Not important | 9.7 (9) | 15 (11) |
| Important but not critical | 41.9 (39) | 57 (41) |
| Critical | 48.4 (45) | 28 (20) |
| **Anxiety** |  |  |
| Not important | 31.2 (29) | 32 (23) |
| Important but not critical | 49.5 (46) | 58 (42) |
| Critical | 19.4 (18) | 10 (7) |
| **Sleep disturbance** |  |  |
| Not important | 23.7 (22) | 29 (21) |
| Important but not critical | 52.7 (49) | 62 (45) |
| Critical | 23.7 (22) | 8 (6) |
| **Self esteem** |  |  |
| Not important | 29.0 (27) | 28 (20) |
| Important but not critical | 54.8 (51) | 62 (45) |
| Critical | 16.1 (15) | 10 (7) |
| **Dietary intake** |  |  |
| Not important | 0 (0) | n/a |
| Important but not critical | 17.2 (23) | n/a |
| Critical | 82.8 (77) | n/a |
| **Appetite** |  |  |
| Not important | 2.2 (2) | 6 (4) |
| Important but not critical | 38.7 (36) | 31 (22) |
| Critical | 59.1 (55) | 64 (46) |
| **Hydration status** |  |  |
| Not important | 7.5 (7) | 4 (3) |
| Important but not critical | 29.0 (27) | 47 (34) |
| Critical | 63.4 (59) | 49 (35) |
| **Eating behaviour** |  |  |
| Not important | 12.9 (12) | 10 (7) |
| Important but not critical | 44.1 (41) | 50 (36) |
| Critical | 43.0 (40) | 40 (29) |
| **Energy requirements** |  |  |
| Not important | 12.9 (12) | 10 (7) |
| Important but not critical | 40.9 (38) | 58 (42) |
| Critical | 46.2 (43) | 32 (23) |
| **Blood marker(s)** |  |  |
| Not important | 28.0 (26) | 22 (16) |
| Important but not critical | 40.9 (38) | 53 (38) |
| Critical | 31.2 (29) | 25 (18) |
| **Nitrogen balance** |  |  |
| Not important | 40.9 (38) | 44 (32) |
| Important but not critical | 48.4 (45) | 49 (35) |
| Critical | 10.8 (10) | 7 (5) |
| **Muscle mass** |  |  |
| Not important | 6.5 (6) | 0 (0) |
| Important but not critical | 28.0 (26) | 18.1 (13) |
| Critical | 65.6 (61) | 82 (59) |
| **Muscle strength** |  |  |
| Not important | 2.2 (2) | n/a |
| Important but not critical | 16.1 (15) | n/a |
| Critical | 81.7 (76) | n/a |
| **Functional performance** |  |  |
| Not important | 3.2 (3) | n/a |
| Important but not critical | 11.8 (11) | n/a |
| Critical | 84.9 (79) | n/a |
| **Functional limitations** |  |  |
| Not important | 1.1 (1) | n/a |
| Important but not critical | 26.9 (25) | n/a |
| Critical | 72.0 (67) | n/a |
| **Participation in social roles and activities** |  |  |
| Not important | 8.6 (8) | 8 (6) |
| Important but not critical | 40.9 (38) | 44 (32) |
| Critical | 50.5 (47) | 47 (34) |
| **Peak expiratory flow** |  |  |
| Not important | 48.4 (45) | 39 (28) |
| Important but not critical | 38.7 (36) | 50 (36) |
| Critical | 12.9 (12) | 11 (8) |
| **Bone health** |  |  |
| Not important | 26.9 (25) | 14 (10) |
| Important but not critical | 46.2 (43) | 64 (46) |
| Critical | 26.9 (25) | 22 (16) |
| **Acceptability of the intervention** |  |  |
| Not important | 2.2 (2) | n/a |
| Important but not critical | 19.4 (18) | n/a |
| Critical | 78.5 (73) | n/a |
| **Adverse events** |  |  |
| Not important | 7.5 (7) | n/a |
| Important but not critical | 37.6 (35) | n/a |
| Critical | 54.8 (51) | n/a |
| **Physical activity** |  |  |
| Not important | n/a | 3 (2) |
| Important but not critical | n/a | 31 (22) |
| Critical | n/a | 67 (48) |

Note: Adverse events were excluded for the 2^nd^ round and physical activity was included. Malnutrition status, body weight or BMI, functional performance, dietary intake, muscle strength, functional limitations, quality of life and acceptability of intervention met the inclusion criteria in the 1^st^ round and the 2^nd^ round only asked if participants agreed to their inclusion in the core outcome set. BMI, body mass index; n/a, not applicable.

**Table S3.** Rating of outcomes (not important, important but not critical and critical) for the 1^st^ Delphi Survey round per setting and per follow-up duration of nutritional interventions.

|  | **Community % (n)** | **Hospital % (n)** | **Long-term care % (n)** | **<12 weeks % (n)** | **≥12 weeks % (n)** | ***P^set^*** | ***p^fu^*** |
| --- | --- | --- | --- | --- | --- | --- | --- |
| **Number of participants** | 35.5 (33) | 54.8 (51) | 9.7 (9) | 48.4 (45) | 51.6 (48) |  |  |
| **Body weight/ BMI** |  |  |  |  |  | 0.503 | 0.138 |
| Not important | 9.1 (3) | 7.8 (4) | 0.0 (0) | 4.4 (2) | 10.4 (5) |  |  |
| Important but not critical | 18.2 (6) | 19.6 (10) | 0.0 (0) | 24.4 (11) | 10.4 (5) |  |  |
| Critical | 72.7 (24) | 72.5 (37) | 100.0 (9) | 71.1 (32) | 79.2 (38) |  |  |
| **Body circumference(s)** |  |  |  |  |  | 0.834 | 0.478 |
| Not important | 18.2 (6) | 25.5 (13) | 22.2 (2) | 22.2 (10) | 22.9 (11) |  |  |
| Important but not critical | 45.5 (15) | 39.2 (20) | 55.6 (5) | 48.9 (22) | 37.5 (18) |  |  |
| Critical | 36.4 (12) | 35.3 (18) | 22.2 (2) | 28.9 (13) | 39.6 (19) |  |  |
| **Skinfold(s)** |  |  |  |  |  | 0.840 | 0.636 |
| Not important | 39.4 (13) | 45.1 (23) | 44.4 (4) | 44.4 (20) | 41.7 (20) |  |  |
| Important but not critical | 48.5 (16) | 45.1 (23) | 55.6 (5) | 48.9 (22) | 45.8 (22) |  |  |
| Critical | 12.1 (4) | 9.8 (5) | 0.0 (0) | 6.7 (3) | 12.5 (6) |  |  |
| **Malnutrition status** |  |  |  |  |  | 0.727 | 0.511 |
| Not important | 0.0 (0) | 3.9 (2) | 0.0 (0) | 2.2 (1) | 2.1 (1) |  |  |
| Important but not critical | 12.1 (4) | 7.8 (4) | 11.1 (1) | 13.3 (6) | 6.2 (3) |  |  |
| Critical | 87.9 (29) | 88.2 (45) | 88.9 (8) | 84.4 (38) | 91.7 (44) |  |  |
| **Falls** |  |  |  |  |  | 0.362 | 0.550 |
| Not important | 6.1 (2) | 2.0 (1) | 11.1 (1) | 6.7 (3) | 2.1 (1) |  |  |
| Important but not critical | 39.4 (13) | 25.5 (13) | 22.2 (2) | 28.9 (13) | 31.2 (15) |  |  |
| Critical | 54.5 (18) | 72.5 (37) | 66.7 (6) | 64.4 (29) | 66.7 (32) |  |  |
| **Frailty** |  |  |  |  |  | 0.679 | 0.943 |
| Not important | 3.0 (1) | 3.9 (2) | 11.1 (1) | 4.4 (2) | 4.2 (2) |  |  |
| Important but not critical | 18.2 (6) | 25.5 (13) | 11.1 (1) | 20.0 (9) | 22.9 (11) |  |  |
| Critical | 78.8 (26) | 70.6 (36) | 77.8 (7) | 75.6 (34) | 72.9 (35) |  |  |
| **Mortality** |  |  |  |  |  | 0.087 | 0.550 |
| Not important | 9.1 (3) | 2.0 (1) | 0.0 (0) | 6.7 (3) | 2.1 (1) |  |  |
| Important but not critical | 42.4 (14) | 21.6 (11) | 33.3 (3) | 28.9 (13) | 31.2 (15) |  |  |
| Critical | 48.5 (16) | 76.5 (39) | 66.7 (6) | 64.4 (29) | 66.7 (32) |  |  |
| **Healthcare use** |  |  |  |  |  | 0.638 | 0.086 |
| Not important | 3.0 (1) | 9.8 (5) | 0.0 (0) | 8.9 (4) | 4.2 (2) |  |  |
| Important but not critical | 42.4 (14) | 45.1 (23) | 44.4 (4) | 53.3 (24) | 35.4 (17) |  |  |
| Critical | 54.5 (18) | 45.1 (23) | 55.6 (5) | 37.8 (17) | 60.4 (29) |  |  |
| **Healthcare costs** |  |  |  |  |  | 0.860 | 0.848 |
| Not important | 6.1 (2) | 13.7 (7) | 11.1 (1) | 8.9 (4) | 12.5 (6) |  |  |
| Important but not critical | 51.5 (17) | 45.1 (23) | 44.4 (4) | 48.9 (22) | 45.8 (22) |  |  |
| Critical | 42.4 (14) | 41.2 (21) | 44.4 (4) | 42.2 (19) | 41.7 (20) |  |  |
| **Complications** |  |  |  |  |  | 0.014 | 0.515 |
| Not important | 9.1 (3) | 2.0 (1) | 22.2 (2) | 8.9 (4) | 4.2 (2) |  |  |
| Important but not critical | 51.5 (17) | 27.5 (14) | 22.2 (2) | 31.1 (14) | 39.6 (19) |  |  |
| Critical | 39.4 (13) | 70.6 (36) | 55.6 (5) | 60.0 (27) | 56.2 (27) |  |  |
| **Health status** |  |  |  |  |  | 0.633 | 0.284 |
| Not important | 3.0 (1) | 0.0 (0) | 0.0 (0) | 0.0 (0) | 2.1 (1) |  |  |
| Important but not critical | 27.3 (9) | 37.3 (19) | 33.3 (3) | 40.0 (18) | 27.1 (13) |  |  |
| Critical | 69.7 (23) | 62.7 (32) | 66.7 (6) | 60.0 (27) | 70.8 (34) |  |  |
| **Dysphagia severity** |  |  |  |  |  | 0.008 | 0.244 |
| Not important | 33.3 (11) | 9.8 (5) | 0.0 (0) | 11.1 (5) | 22.9 (11) |  |  |
| Important but not critical | 30.3 (10) | 37.3 (19) | 11.1 (1) | 31.1 (14) | 33.3 (16) |  |  |
| Critical | 36.4 (12) | 52.9 (27) | 88.9 (8) | 57.8 (26) | 43.8 (21) |  |  |
| **Fatigue** |  |  |  |  |  | 0.120 | 0.300 |
| Not important | 6.1 (2) | 21.6 (11) | 22.2 (2) | 22.2 (10) | 10.4 (5) |  |  |
| Important but not critical | 60.6 (20) | 35.3 (18) | 55.6 (5) | 42.2 (19) | 50.0 (24) |  |  |
| Critical | 33.3 (11) | 43.1 (22) | 22.2 (2) | 35.6 (16) | 39.6 (19) |  |  |
| **Weakness** |  |  |  |  |  | 0.166 | 0.065 |
| Not important | 0.0 (0) | 11.8 (6) | 22.2 (2) | 15.6 (7) | 2.1 (1) |  |  |
| Important but not critical | 54.5 (18) | 41.2 (21) | 44.4 (4) | 44.4 (20) | 47.9 (23) |  |  |
| Critical | 45.5 (15) | 47.1 (24) | 33.3 (3) | 40.0 (18) | 50.0 (24) |  |  |
| **Self perceived health** |  |  |  |  |  | 0.072 | 0.945 |
| Not important | 3.0 (1) | 9.8 (5) | 0.0 (0) | 6.7 (3) | 6.2 (3) |  |  |
| Important but not critical | 33.3 (11) | 56.9 (29) | 44.4 (4) | 48.9 (22) | 45.8 (22) |  |  |
| Critical | 63.6 (21) | 33.3 (17) | 55.6 (5) | 44.4 (20) | 47.9 (23) |  |  |
| **Pain** |  |  |  |  |  | 0.524 | 0.916 |
| Not important | 18.2 (6) | 23.5 (12) | 33.3 (3) | 24.4 (11) | 20.8 (10) |  |  |
| Important but not critical | 54.5 (18) | 37.3 (19) | 33.3 (3) | 42.2 (19) | 43.8 (21) |  |  |
| Critical | 27.3 (9) | 39.2 (20) | 33.3 (3) | 33.3 (15) | 35.4 (17) |  |  |
| **Quality of life** |  |  |  |  |  | 0.511 | 0.446 |
| Not important | 0.0 (0) | 2.0 (1) | 0.0 (0) | 2.2 (1) | 0.0 (0) |  |  |
| Important but not critical | 21.2 (7) | 21.6 (11) | 0.0 (0) | 22.2 (10) | 16.7 (8) |  |  |
| Critical | 78.8 (26) | 76.5 (39) | 100.0 (9) | 75.6 (34) | 83.3 (40) |  |  |
| **Cognitive status** |  |  |  |  |  | 0.147 | 0.864 |
| Not important | 15.2 (5) | 7.8 (4) | 0.0 (0) | 11.1 (5) | 8.3 (4) |  |  |
| Important but not critical | 48.5 (16) | 29.4 (15) | 44.4 (4) | 35.6 (16) | 39.6 (19) |  |  |
| Critical | 36.4 (12) | 62.7 (32) | 55.6 (5) | 53.3 (24) | 52.1 (25) |  |  |
| **Depression** |  |  |  |  |  | 0.021 | 0.621 |
| Not important | 6.1 (2) | 13.7 (7) | 0.0 (0) | 6.7 (3) | 12.5 (6) |  |  |
| Important but not critical | 63.6 (21) | 29.4 (15) | 33.3 (3) | 44.4 (20) | 39.6 (19) |  |  |
| Critical | 30.3 (10) | 56.9 (29) | 66.7 (6) | 48.9 (22) | 47.9 (23) |  |  |
| **Anxiety** |  |  |  |  |  | 0.309 | 0.340 |
| Not important | 36.4 (12) | 25.5 (13) | 44.4 (4) | 37.8 (17) | 25.0 (12) |  |  |
| Important but not critical | 54.5 (18) | 49.0 (25) | 33.3 (3) | 42.2 (19) | 56.2 (27) |  |  |
| Critical | 9.1 (3) | 25.5 (13) | 22.2 (2) | 20.0 (9) | 18.8 (9) |  |  |
| **Sleep disturbance** |  |  |  |  |  | 0.151 | 0.123 |
| Not important | 36.4 (12) | 15.7 (8) | 22.2 (2) | 31.1 (14) | 16.7 (8) |  |  |
| Important but not critical | 51.5 (17) | 54.9 (28) | 44.4 (4) | 42.2 (19) | 62.5 (30) |  |  |
| Critical | 12.1 (4) | 29.4 (15) | 33.3 (3) | 26.7 (12) | 20.8 (10) |  |  |
| **Self esteem** |  |  |  |  |  | 0.223 | 0.157 |
| Not important | 30.3 (10) | 25.5 (13) | 44.4 (4) | 28.9 (13) | 29.2 (14) |  |  |
| Important but not critical | 60.6 (20) | 56.9 (29) | 22.2 (2) | 62.2 (28) | 47.9 (23) |  |  |
| Critical | 9.1 (3) | 17.6 (9) | 33.3 (3) | 8.9 (4) | 22.9 (11) |  |  |
| **Dietary intake** |  |  |  |  |  | 0.876 | 0.677 |
| Not important | 0 (0) | 0 (0) | (0) | 0 (0) | 0 (0) |  |  |
| Important but not critical | 15.2 (5) | 17.6 (9) | 22.2 (2) | 20 (9) | 14.6 (7) |  |  |
| Critical | 84.8 (28) | 82.4 (42) | 77.8 (7) | 80.0 (36) | 85.4 (41) |  |  |
| **Appetite** |  |  |  |  |  | 0.390 | 0.791 |
| Not important | 0.0 (0) | 3.9 (2) | 0.0 (0) | 2.2 (1) | 2.1 (1) |  |  |
| Important but not critical | 48.5 (16) | 35.3 (18) | 22.2 (2) | 42.2 (19) | 35.4 (17) |  |  |
| Critical | 51.5 (17) | 60.8 (31) | 77.8 (7) | 55.6 (25) | 62.5 (30) |  |  |
| **Hydration status** |  |  |  |  |  | 0.237 | 0.537 |
| Not important | 6.1 (2) | 9.8 (5) | 0.0 (0) | 4.4 (2) | 10.4 (5) |  |  |
| Important but not critical | 42.4 (14) | 21.6 (11) | 22.2 (2) | 31.1 (14) | 27.1 (13) |  |  |
| Critical | 51.5 (17) | 68.6 (35) | 77.8 (7) | 64.4 (29) | 62.5 (30) |  |  |
| **Eating behaviour** |  |  |  |  |  | 0.260 | 0.325 |
| Not important | 12.1 (4) | 13.7 (7) | 11.1 (1) | 8.9 (4) | 16.7 (8) |  |  |
| Important but not critical | 57.6 (19) | 39.2 (20) | 22.2 (2) | 51.1 (23) | 37.5 (18) |  |  |
| Critical | 30.3 (10) | 47.1 (24) | 66.7 (6) | 40.0 (18) | 45.8 (22) |  |  |
| **Energy requirements** |  |  |  |  |  | 0.087 | 0.984 |
| Not important | 24.2 (8) | 7.8 (4) | 0.0 (0) | 13.3 (6) | 12.5 (6) |  |  |
| Important but not critical | 42.4 (14) | 37.3 (19) | 55.6 (5) | 40.0 (18) | 41.7 (20) |  |  |
| Critical | 33.3 (11) | 54.9 (28) | 44.4 (4) | 46.7 (21) | 45.8 (22) |  |  |
| **Blood marker(s)** |  |  |  |  |  | 0.017 | 0.393 |
| Not important | 42.4 (14) | 17.6 (9) | 33.3 (3) | 26.7 (12) | 29.2 (14) |  |  |
| Important but not critical | 45.5 (15) | 37.3 (19) | 44.4 (4) | 35.6 (16) | 45.8 (22) |  |  |
| Critical | 12.1 (4) | 45.1 (23) | 22.2 (2) | 37.8 (17) | 25.0 (12) |  |  |
| **Nitrogen balance** |  |  |  |  |  | 0.453 | 0.737 |
| Not important | 45.5 (15) | 35.3 (18) | 55.6 (5) | 44.4 (20) | 37.5 (18) |  |  |
| Important but not critical | 39.4 (13) | 54.9 (28) | 44.4 (4) | 46.7 (21) | 50.0 (24) |  |  |
| Critical | 15.2 (5) | 9.8 (5) | 0.0 (0) | 8.9 (4) | 12.5 (6) |  |  |
| **Muscle mass** |  |  |  |  |  | 0.374 | 0.132 |
| Not important | 6.1 (2) | 7.8 (4) | 0.0 (0) | 11.1 (5) | 2.1 (1) |  |  |
| Important but not critical | 27.3 (9) | 23.5 (12) | 55.6 (5) | 31.1 (14) | 25.0 (12) |  |  |
| Critical | 66.7 (22) | 68.6 (35) | 44.4 (4) | 57.8 (26) | 72.9 (35) |  |  |
| **Muscle strength** |  |  |  |  |  | 0.745 | 0.294 |
| Not important | 0.0 (0) | 3.9 (2) | 0.0 (0) | 4.4 (2) | 0.0 (0) |  |  |
| Important but not critical | 15.2 (5) | 15.7 (8) | 22.2 (2) | 17.8 (8) | 14.6 (7) |  |  |
| Critical | 84.8 (28) | 80.4 (41) | 77.8 (7) | 77.8 (35) | 85.4 (41) |  |  |
| **Functional performance** |  |  |  |  |  | 0.327 | 0.093 |
| Not important | 0.0 (0) | 5.9 (3) | 0.0 (0) | 6.7 (3) | 0.0 (0) |  |  |
| Important but not critical | 6.1 (2) | 15.7 (8) | 11.1 (1) | 15.6 (7) | 8.3 (4) |  |  |
| Critical | 93.9 (31) | 78.4 (40) | 88.9 (8) | 77.8 (35) | 91.7 (44) |  |  |
| **Functional limitations** |  |  |  |  |  | 0.771 | 0.517 |
| Not important | 0.0 (0) | 2.0 (1) | 0.0 (0) | 2.2 (1) | 0.0 (0) |  |  |
| Important but not critical | 21.2 (7) | 29.4 (15) | 33.3 (3) | 28.9 (13) | 25.0 (12) |  |  |
| Critical | 78.8 (26) | 68.6 (35) | 66.7 (6) | 68.9 (31) | 75.0 (36) |  |  |
| **Participation in social roles and activities** |  |  |  |  |  | 0.686 | 0.040 |
| Not important | 6.1 (2) | 9.8 (5) | 11.1 (1) | 13.3 (6) | 4.2 (2) |  |  |
| Important but not critical | 39.4 (13) | 45.1 (23) | 22.2 (2) | 48.9 (22) | 33.3 (16) |  |  |
| Critical | 54.5 (18) | 45.1 (23) | 66.7 (6) | 37.8 (17) | 62.5 (30) |  |  |
| **Peak expiratory flow** |  |  |  |  |  | 0.215 | 0.504 |
| Not important | 60.6 (20) | 37.3 (19) | 66.7 (6) | 48.9 (22) | 47.9 (23) |  |  |
| Important but not critical | 30.3 (10) | 47.1 (24) | 22.2 (2) | 42.2 (19) | 35.4 (17) |  |  |
| Critical | 9.1 (3) | 15.7 (8) | 11.1 (1) | 8.9 (4) | 16.7 (8) |  |  |
| **Bone health** |  |  |  |  |  | 0.755 | 0.525 |
| Not important | 27.3 (9) | 23.5 (12) | 44.4 (4) | 31.1 (14) | 22.9 (11) |  |  |
| Important but not critical | 48.5 (16) | 47.1 (24) | 33.3 (3) | 46.7 (21) | 45.8 (22) |  |  |
| Critical | 24.2 (8) | 29.4 (15) | 22.2 (2) | 22.2 (10) | 31.2 (15) |  |  |
| **Acceptability/ adherence of the intervention** |  |  |  |  |  | 0.626 | 0.324 |
| Not important | 0.0 (0) | 3.9 (2) | 0.0 (0) | 4.4 (2) | 0.0 (0) |  |  |
| Important but not critical | 24.2 (8) | 17.6 (9) | 11.1 (1) | 17.8 (8) | 20.8 (10) |  |  |
| Critical | 75.8 (25) | 78.4 (40) | 88.9 (8) | 77.8 (35) | 79.2 (38) |  |  |
| **Adverse events** |  |  |  |  |  | 0.222 | 0.302 |
| Not important | 15.2 (5) | 3.9 (2) | 0.0 (0) | 4.4 (2) | 10.4 (5) |  |  |
| Important but not critical | 36.4 (12) | 35.3 (18) | 55.6 (5) | 44.4 (20) | 31.2 (15) |  |  |
| Critical | 48.5 (16) | 60.8 (31) | 44.4 (4) | 51.1 (23) | 58.3 (28) |  |  |

Note: Main setting refers to the setting where most of the work with older adults with malnutrition and at risk took place. Main follow-up duration refers to the time that was most common/preferred for nutritional interventions in the person’s setting. BMI, body mass index; *p*^fu^, p-value for follow-up duration; *p*^set^, p-value for setting.

**Table S4.** Participants’ rating of the outcomes (not important, important but not critical and critical) for the 1^st^ and 2^nd^ Delphi Survey round by Europe and elsewhere

|  | **Delphi 1^st^ round** % (n) | | **Delphi 2^nd^ round** % (n) | |
| --- | --- | --- | --- | --- |
|  | **Europe** | **Elsewhere** | **Europe** | **Elsewhere** |
| **Number of participants** | 67 | 26 | 54 | 18 |
| **Body weight/ BMI** |  |  |  |  |
| Not important | 7.5 (5) | 7.7 (2) | n/a | n/a |
| Important but not critical | 16.4 (11) | 19.2 (5) | n/a | n/a |
| Critical | 76.1 (51) | 73.1 (19) | n/a | n/a |
| **Body circumference(s)** |  |  |  |  |
| Not important | 26.9 (18) | 11.5 (3) | 22.2 (12) | 5.6 (1) |
| Important but not critical | 35.8 (24) | 61.5 (16) | 55.6 (30) | 72.2 (13) |
| Critical | 37.3 (25) | 26.9 (7) | 22.2 (12) | 22.2 (4) |
| **Skinfold(s)** |  |  |  |  |
| Not important | 46.3 (31) | 34.6 (9) | 38.9 (21) | 27.8 (5) |
| Important but not critical | 43.3 (29) | 57.7 (15) | 51.9 (28) | 66.7 (12) |
| Critical | 10.4 (7) | 7.7 (2) | 9.3 (5) | 5.6 (1) |
| **Malnutrition status** |  |  |  |  |
| Not important | 3.0 (2) | 0.0 (0) | n/a | n/a |
| Important but not critical | 11.9 (8) | 3.8 (1) | n/a | n/a |
| Critical | 85.1 (57) | 96.2 (25) | n/a | n/a |
| **Falls** |  |  |  |  |
| Not important | 6.0 (4) | 0.0 (0) | 7.4 (4) | 0.0 (0) |
| Important but not critical | 31.3 (21) | 26.9 (7) | 38.9 (21) | 16.7 (3) |
| Critical | 62.7 (42) | 73.1 (19) | 53.7 (29) | 83.3 (15) |
| **Frailty** |  |  |  |  |
| Not important | 6.0 (4) | 0.0 (0) | 1.9 (1) | 0.0 (0) |
| Important but not critical | 22.4 (15) | 19.2 (5) | 24.1 (13) | 5.6 (1) |
| Critical | 71.6 (48) | 80.8 (21) | 74.1 (40) | 94.4 (17) |
| **Mortality** |  |  |  |  |
| Not important | 6.0 (4) | 0.0 (0) | 7.4 (4) | 5.6 (1) |
| Important but not critical | 29.9 (20) | 30.8 (8) | 27.8 (15) | 27.8 (5) |
| Critical | 64.2 (43) | 69.2 (18) | 64.8 (35) | 66.7 (12) |
| **Healthcare use** |  |  |  |  |
| Not important | 7.5 (5) | 3.8 (1) | 3.7 (2) | 11.1 (2) |
| Important but not critical | 43.3 (29) | 46.2 (12) | 51.9 (28) | 50.0 (9) |
| Critical | 49.3 (33) | 50.0 (13) | 44.4 (24) | 38.9 (7) |
| **Healthcare costs** |  |  |  |  |
| Not important | 7.5 (5) | 19.2 (5) | 9.3 (5) | 0.0 (0) |
| Important but not critical | 52.2 (35) | 34.6 (9) | 51.9 (28) | 55.6 (10) |
| Critical | 40.3 (27) | 46.2 (12) | 38.9 (21) | 44.4 (8) |
| **Complications** |  |  |  |  |
| Not important | 4.5 (3) | 11.5 (3) | 3.7 (2) | 5.6 (1) |
| Important but not critical | 34.3 (23) | 38.5 (10) | 38.9 (21) | 22.2 (4) |
| Critical | 61.2 (41) | 50.0 (13) | 57.4 (31) | 72.2 (13) |
| **Health status** |  |  |  |  |
| Not important | 1.5 (1) | 0.0 (0) | 3.7 (2) | 5.6 (1) |
| Important but not critical | 32.8 (22) | 34.6 (9) | 31.5 (17) | 22.2 (4) |
| Critical | 65.7 (44) | 65.4 (17) | 64.8 (35) | 72.2 (13) |
| **Dysphagia severity** |  |  |  |  |
| Not important | 20.9 (14) | 7.7 (2) | 13.0 (7) | 16.7 (3) |
| Important but not critical | 31.3 (21) | 34.6 (9) | 40.7 (22) | 27.8 (5) |
| Critical | 47.8 (32) | 57.7 (15) | 46.3 (25) | 55.6 (10) |
| **Fatigue** |  |  |  |  |
| Not important | 19.4 (13) | 7.7 (2) | 11.1 (6) | 5.6 (1) |
| Important but not critical | 46.3 (31) | 46.2 (12) | 57.4 (31) | 55.6 (10) |
| Critical | 34.3 (23) | 46.2 (12) | 31.5 (17) | 38.9 (7) |
| **Weakness** |  |  |  |  |
| Not important | 10.4 (7) | 3.8 (1) | 14.8 (8) | 0.0 (0) |
| Important but not critical | 47.8 (32) | 42.3 (11) | 46.3 (25) | 50.0 (9) |
| Critical | 41.8 (28) | 53.8 (14) | 38.9 (21) | 50.0 (9) |
| **Self perceived health** |  |  |  |  |
| Not important | 7.5 (5) | 3.8 (1) | 7.4 (4) | 11.1 (2) |
| Important but not critical | 53.7 (36) | 30.8 (8) | 57.4 (31) | 44.4 (8) |
| Critical | 38.8 (26) | 65.4 (17) | 35.2 (19) | 44.4 (8) |
| **Pain** |  |  |  |  |
| Not important | 23.9 (16) | 19.2 (5) | 25.9 (14) | 16.7 (3) |
| Important but not critical | 41.8 (28) | 46.2 (12) | 48.1 (26) | 61.1 (11) |
| Critical | 34.3 (23) | 34.6 (9) | 25.9 (14) | 22.2 (4) |
| **Quality of life** |  |  |  |  |
| Not important | 1.5 (1) | 0.0 (0) | n/a | n/a |
| Important but not critical | 20.9 (14) | 15.4 (4) | n/a | n/a |
| Critical | 77.6 (52) | 84.6 (22) | n/a | n/a |
| **Cognitive status** |  |  |  |  |
| Not important | 13.4 (9) | 0.0 (0) | 14.8 (8) | 16.7 (3) |
| Important but not critical | 35.8 (24) | 42.3 (11) | 40.7 (22) | 44.4 (8) |
| Critical | 50.7 (34) | 57.7 (15) | 44.4 (24) | 38.9 (7) |
| **Depression** |  |  |  |  |
| Not important | 11.9 (8) | 3.8 (1) | 14.8 (8) | 16.7 (3) |
| Important but not critical | 46.3 (31) | 30.8 (8) | 59.3 (32) | 50.0 (9) |
| Critical | 41.8 (28) | 65.4 (17) | 25.9 (14) | 33.3 (6) |
| **Anxiety** |  |  |  |  |
| Not important | 37.3 (25) | 15.4 (4) | 37.0 (20) | 16.7 (3) |
| Important but not critical | 44.8 (30) | 61.5 (16) | 53.7 (29) | 72.2 (13) |
| Critical | 17.9 (12) | 23.1 (6) | 9.3 (5) | 11.1 (2) |
| **Sleep disturbance** |  |  |  |  |
| Not important | 28.4 (19) | 11.5 (3) | 33.3 (18) | 16.7 (3) |
| Important but not critical | 49.3 (33) | 61.5 (16) | 59.3 (32) | 72.2 (13) |
| Critical | 22.4 (15) | 26.9 (7) | 7.4 (4) | 11.1 (2) |
| **Self esteem** |  |  |  |  |
| Not important | 34.3 (23) | 15.4 (4) | 31.5 (17) | 16.7 (3) |
| Important but not critical | 50.7 (34) | 65.4 (17) | 57.4 (31) | 77.8 (14) |
| Critical | 14.9 (10) | 19.2 (5) | 11.1 (6) | 5.6 (1) |
| **Dietary intake** |  |  |  |  |
| Not important | 0 (0) | 0 (0) | n/a | n/a |
| Important but not critical | 14.9 (10) | 23.1 (6) | n/a | n/a |
| Critical | 85.1 (57) | 76.9 (20) | n/a | n/a |
| **Appetite** |  |  |  |  |
| Not important | 1.5 (1) | 3.8 (1) | 7.4 (4) | 0.0 (0) |
| Important but not critical | 37.3 (25) | 42.3 (11) | 27.8 (15) | 38.9 (7) |
| Critical | 61.2 (41) | 53.8 (14) | 64.8 (35) | 61.1 (11) |
| **Hydration status** |  |  |  |  |
| Not important | 7.5 (5) | 7.7 (2) | 5.6 (3) | 0.0 (0) |
| Important but not critical | 28.4 (19) | 30.8 (8) | 44.4 (24) | 55.6 (10) |
| Critical | 64.2 (43) | 61.5 (16) | 50.0 (27) | 44.4 (8) |
| **Eating behaviour** |  |  |  |  |
| Not important | 13.4 (9) | 11.5 (3) | 11.1 (6) | 5.6 (1) |
| Important but not critical | 40.3 (27) | 53.8 (14) | 44.4 (24) | 66.7 (12) |
| Critical | 46.3 (31) | 34.6 (9) | 44.4 (24) | 27.8 (5) |
| **Energy requirements** |  |  |  |  |
| Not important | 16.4 (11) | 3.8 (1) | 11.1 (6) | 5.6 (1) |
| Important but not critical | 41.8 (28) | 38.5 (10) | 61.1 (33) | 50.0 (9) |
| Critical | 41.8 (28) | 57.7 (15) | 27.8 (15) | 44.4 (8) |
| **Blood marker(s)** |  |  |  |  |
| Not important | 31.3 (21) | 19.2 (5) | 24.1 (13) | 16.7 (3) |
| Important but not critical | 34.3 (23) | 57.7 (15) | 48.1 (26) | 66.7 (12) |
| Critical | 34.3 (23) | 23.1 (6) | 27.8 (15) | 16.7 (3) |
| **Nitrogen balance** |  |  |  |  |
| Not important | 43.3 (29) | 34.6 (9) | 48.1 (26) | 33.3 (6) |
| Important but not critical | 46.3 (31) | 53.8 (14) | 44.4 (24) | 61.1 (11) |
| Critical | 10.4 (7) | 11.5 (3) | 7.4 (4) | 5.6 (1) |
| **Muscle mass** |  |  |  |  |
| Not important | 6.0 (4) | 7.7 (2) | 0 | 0 |
| Important but not critical | 26.9 (18) | 30.8 (8) | 22.2 (12) | 5.6 (1) |
| Critical | 67.2 (45) | 61.5 (16) | 77.8 (42) | 94.4 (17) |
| **Muscle strength** |  |  |  |  |
| Not important | 3.0 (2) | 0.0 (0) | n/a | n/a |
| Important but not critical | 16.4 (11) | 15.4 (4) | n/a | n/a |
| Critical | 80.6 (54) | 84.6 (22) | n/a | n/a |
| **Functional performance** |  |  |  |  |
| Not important | 4.5 (3) | 0.0 (0) | n/a | n/a |
| Important but not critical | 11.9 (8) | 11.5 (3) | n/a | n/a |
| Critical | 83.6 (56) | 88.5 (23) | n/a | n/a |
| **Functional limitations** |  |  |  |  |
| Not important | 1.5 (1) | 0.0 (0) | n/a | n/a |
| Important but not critical | 31.3 (21) | 15.4 (4) | n/a | n/a |
| Critical | 67.2 (45) | 84.6 (22) | n/a | n/a |
| **Participation in social roles and activities** |  |  |  |  |
| Not important | 3.0 (2) | 23.1 (6) | 9.3 (5) | 5.6 (1) |
| Important but not critical | 41.8 (28) | 38.5 (10) | 40.7 (22) | 55.6 (10) |
| Critical | 55.2 (37) | 38.5 (10) | 50.0 (27) | 38.9 (7) |
| **Peak expiratory flow** |  |  |  |  |
| Not important | 50.7 (34) | 42.3 (11) | 42.6 (23) | 27.8 (5) |
| Important but not critical | 34.3 (23) | 50.0 (13) | 46.3 (25) | 61.1 (11) |
| Critical | 14.9 (10) | 7.7 (2) | 11.1 (6) | 11.1 (2) |
| **Bone health** |  |  |  |  |
| Not important | 28.4 (19) | 23.1 (6) | 16.7 (9) | 5.6 (1) |
| Important but not critical | 47.8 (32) | 42.3 (11) | 63.0 (34) | 66.7 (12) |
| Critical | 23.9 (16) | 34.6 (9) | 20.4 (11) | 27.8 (5) |
| **Acceptability of the intervention** |  |  |  |  |
| Not important | 1.5 (1) | 3.8 (1) | n/a | n/a |
| Important but not critical | 20.9 (14) | 15.4 (4) | n/a | n/a |
| Critical | 77.6 (52) | 80.8 (21) | n/a | n/a |
| **Adverse events** |  |  |  |  |
| Not important | 7.5 (5) | 7.7 (2) | n/a | n/a |
| Important but not critical | 41.8 (28) | 26.9 (7) | n/a | n/a |
| Critical | 50.7 (34) | 65.4 (17) | n/a | n/a |
| **Physical activity** |  |  |  |  |
| Not important | n/a | n/a | 3.7 (2) | 0.0 (0) |
| Important but not critical | n/a | n/a | 33.3 (18) | 22.2 (4) |
| Critical | n/a | n/a | 63.0 (34) | 77.8 (14) |
